# Supplementary material for: Persistent Human KIT Receptor Signaling Disposes Murine Placenta to Premature Differentiation Resulting in Severely Disrupted Placental Structure and Functionality
Source: Int J Mol Sci. 2020 Jul 31;21(15):5503. doi: 10.3390/ijms21155503 (PMC7432075; doi:10.3390/ijms21155503)
Supplement: Supplementary file 1 [file ijms-21-05503-s001.pdf]

Persistent Human KIT Receptor Signaling Disposes Murine Placenta to Premature Differentiation Resulting in Severely Disrupted Placental Structure and Functionality

Franziska Kaiser [1,†], Julia Hartweg [1,2,†], Selina Jansky [1,3,4], Natalie Pelusi [1,5], Caroline Kubaczka [1,6], Neha Sharma [1,7], Dominik Nitsche [1,8], Jan Langkabel [1] and Hubert Schorle [1,*]

Supplementary Information

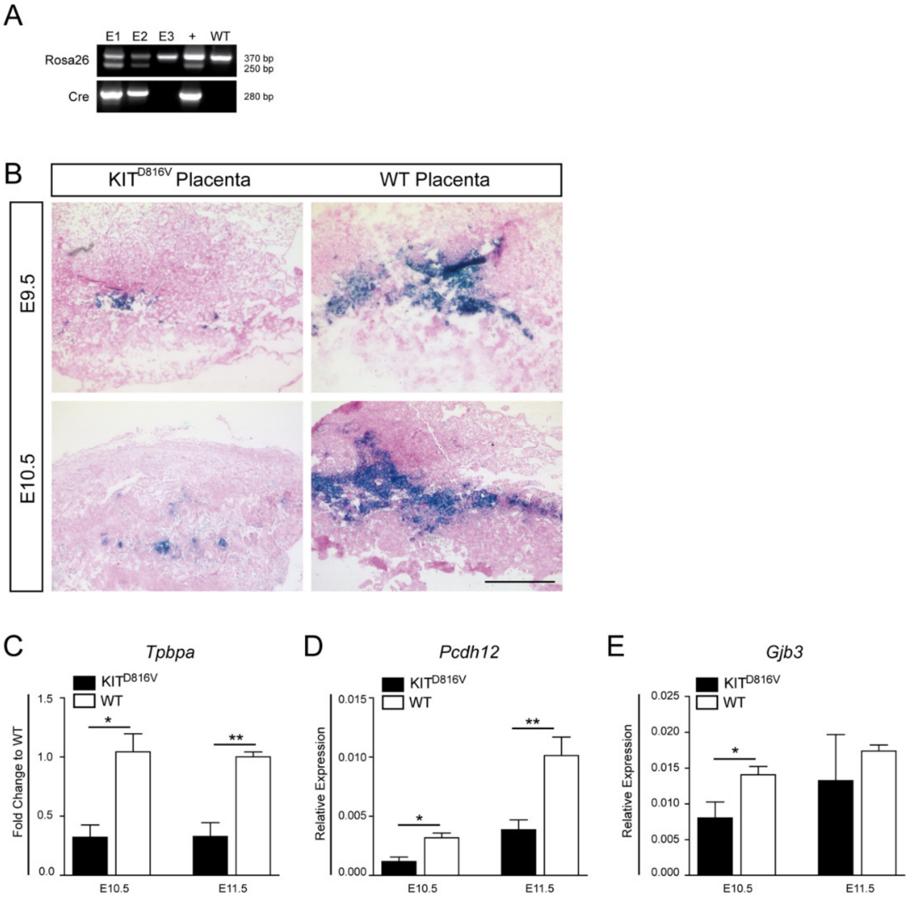

Supplementary Figure S1. Spongiotrophoblast marker TPBPA reduced in KIT^{D816V} placentas: (**A**) Rosa26-KIT^{D816V}-GFP transgene were mated with Deleter-Cre mice. Representative result of genotyping PCR for ROSA26-KIT^{D816V} and Cre transgenes using specific primers. Lines 1 and 2 show KIT^{D816V} and Cre-transgene positive embryos, whereas lane 3 represents a wildtype embryo. Lanes 4 and 5 represent positive and negative controls. (**B**) *In situ* hybridization of TPBPA on cryosections of KIT^{D816V} and control placentas at E9.5 and E10.5 using specific probe for TPBPA: Counterstaining was performed with nuclear fast red. Scale bar represents 500 µm. Two biological replicates were performed. (**C**) qRT-PCR analysis of *Tpbpa* expression in KIT^{D816V} and WT placentas at E10.5 and E11.5, (**D**) qRT-PCR analysis of *Pcdh12* expression in KIT^{D816V} and WT placentas at E10.5 and E11.5, and (**E**) qRT-PCR analysis of *Gjb3* expression in KIT^{D816V} and WT placentas at E10.5 and E11.5: RNA was obtained from three biological replicates. Expression was normalized to the housekeeping gene *Gapdh*. Bars display mean value ± SD. Significance was determined by unpaired t-test and indicated with $*p < 0.05$ and $**p < 0.01$.

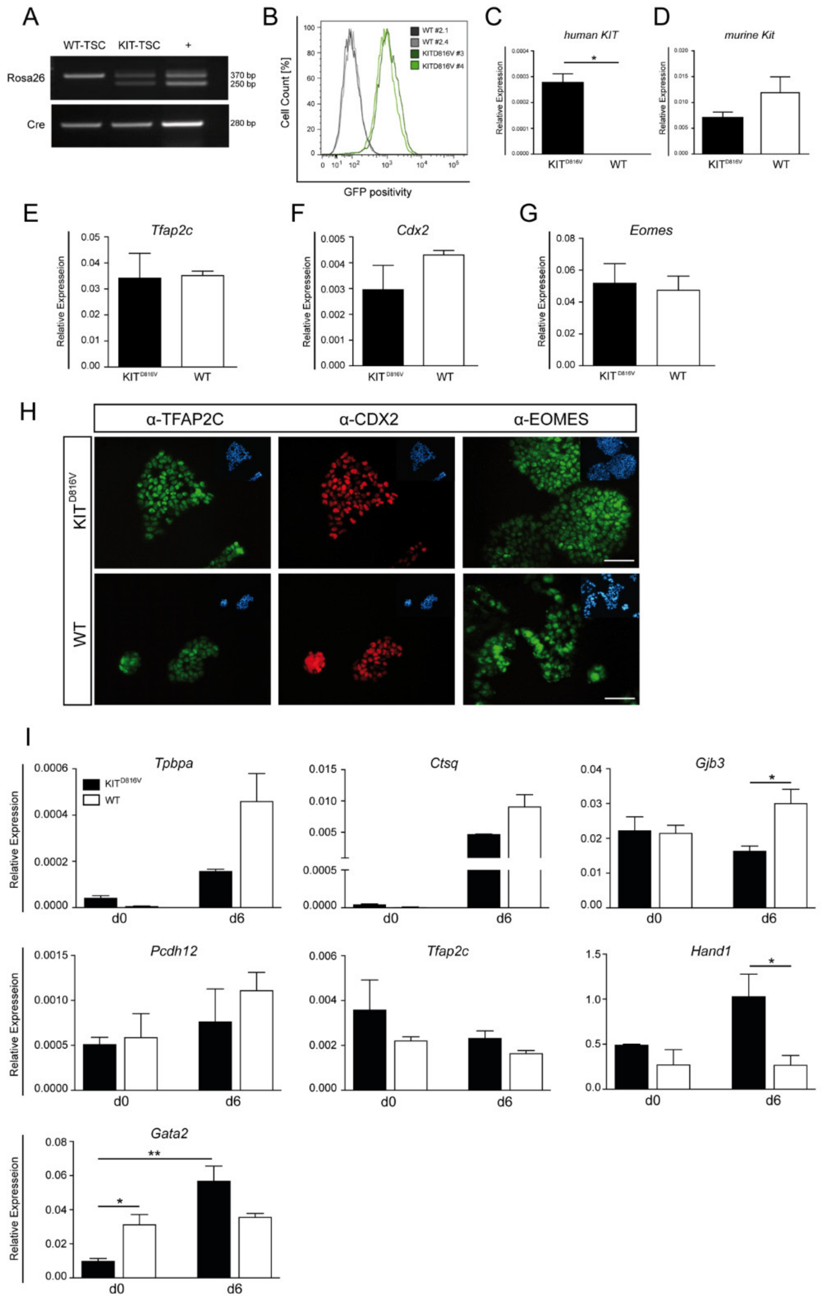

Supplementary Figure S2. Validation of KIT^{D816V}-TSC: (**A**) Representative genotyping PCR on newly derived TSC lines. The results show identification of WT-TSC (lane 1) and KIT^{D816V}-TSC (lane 2) in comparison to positive control (lane 3). (**B**) Flow cytometry analysis of GFP-positive cells on TSC lines derived from mating of ROSA26-KIT^{D816V}-GFP mice with Deleter-Cre mice. Two KIT^{D816V}-TSC and two WT lines were identified. (**C**) qRT-PCR for transgene expression of *human KIT* in KIT^{D816V}- and WT-TSC and (**D**) qRT-PCR for endogenous expression of *murine Kit* in KIT^{D816V}- and WT-TSC: RNA was obtained from two KIT^{D816V}- and two WT-TSC lines (biological replicates = 2); expression is normalized to the housekeeping gene *Gapdh.* Bars display mean value ± SD. Significance was determined by unpaired t-test and indicated with $*p < 0.05$. (**E–G**) qRT-PCR analysis of TSC-specific

markers *Tfap2c*, *Cdx2*, and *Eomes* in RNA isolated from KIT^{D816V}-TSC and WT-TSC. RNA was obtained from two KIT^{D816V}- and two WT-TSC lines (biological replicates = 2). Expression is normalized to the housekeeping gene *Gapdh.* Bars display mean value ± SD. (**H**) Representative immunofluorescence staining against TFAP2C, CDX2, and EOMES in KIT^{D816V}-TSC and WT-TSC. Inlets represent Hoechst staining. Scale bar: 100 µm. (I) qRT-PCR analysis of endogenous expression of *Tpbpa, Ctsq, Gjb3, Pcdh12, Tfap2c, Mash2, Hand1*, and *Gata2* in KIT^{D816V}-TSC line #4 and WT-TSC line 2.1 in undifferentiated states and after culture under differentiation conditions for 6 days. RNA was obtained from three biological replicates. Expression was normalized to the housekeeping gene *Gapdh*; data is represented by mean value ± SD; Significance was determined by unpaired t-test and indicated with *$p < 0.05$ and **$p < 0.01$.

Supplementary Tables

Supplementary Table 1: Genotyping Primers.

Target Gene	Primer	Primer Sequence
Cre Del	Forward (5’->3’)	CGCATAACCAGTGAAA CAGCAT
	Reverse (5’->3’)	GAAAGTCGAGTAGGCG TGTACG
Rosa26	Forward (5’->3’) WT	CTCCCAAAGTCGCTGC TCTGAGT
	Reverse (5’->3’) WT	CCCATTTTCCTTATTTG CCCC
	Reverse (5’->3’) SA	GACATCATCAAGGAAA CCCT

Supplementary Table 2: Antibodies.

Antibody	Dilution	Company	Catalogue #
2A-peptide	1:1000	Merck	MABS2005
Anti-Dioxigenin-AP, Fab fragments	1:2000	Roche	11093274910
BrightVision+ Poly-AP-Anti Mouse/Rabbit IgG Biotin-free	Ready-to-use	ImmunoLogic	DPVB-AP
CD31	1:50	Dianova	SZ31
CDX2	1:200	BioCare Medical	CM226B
cKIT	1:200	Santa Cruz	sc-168
EOMES	1:500	abcam	ab23345

Goat-anti-rabbit Alexa 488	1:500	Invitrogen Life Technologie Inc.	A27034
Goat-anti-rabbit Alexa 594	1:500	Invitrogen Life Technologie Inc.	A-11037
Goat-anti-rabbit HRP	1:2000	DAKO, Agilent Technologies	P0048
KI-67	1:100	Abcam	S86
pAkt	1:2000	Cell Signaling Technology	4060
pErk1/2	1:1000	Cell Signaling Technology	4370
Rabbit-anti-mouse Alexa 488	1:500	Invitrogen Life Technologie Inc.	A27023
Rabbit-anti-mouse HRP	1:1000	DAKO, Agilent Technologies	P0260
TFAP2C	1:300	Santa Cruz	sc-8977
β-ACTIN	1:50000	Sigma Aldrich	a5441

Supplementary Table 3: qRT-PCR Primers.

Target Gene	Forward (5'->3')	Reverse (5'->3')
Human KIT	TTCTTACCAGGTGGCAAAGG	CCTAAAGAGAACAGCTCCCAAA
Cdx2	TCCTGCTGACTGCTTTCTGA	CCCTTCCTGATTTGTGGAGA
Ctsq	GAGGCAGTAGTGGTCATCCC	CAGTACTTCTTCCTCCGGACT
Eomes	CCTGGTGGTGTTTTGTTGTG	TTTAATAGCACCGGGCACTC

Gapdh	ACCACAGTCCATGCCATCAC	TCCACCACCCTGTTGCTGTA
Gata2	CCTCCAGCTTCACCCCTAAG	ACAGGCATTGCACAGGTAGT
Gcm1	TGCACTGCCCGGCAAGAGCA	TCTCCTTCTTCCTCTTCCTC
Gjb3	CTCCTCTGCTGTGGGTCTTG	ATGCCGTGGAGTACTGGTT
Hand1	GAACTCAAAAAGACGGATGGTGG	CGCCCAGACTTGCTGAGG
Mash2	GGTGACTCCTGGTGGACCTA	TCCGGAAGATGGAAGATGTC
Mouse Kit	AGAAGCAGATCTCGGACAGC	CGTAAAGGCGGAATCACAGT
Pcdh12	CTCCTGTCCAGCAAATCTCC	TCTGCTTGACCACTAGGCTTG
Pl1	TGGAGCCTACATTGTGGTGG	TGGCAGTTGGTTTGGAGGA
Pl2	CCAACGTGTGATTGTGGTGT	TGCCACCATGTGTTTCAGAG
Plf	TGCTCCTGGATACTGCTCCTA	GGCTTGTTCCTTGTTTTCTGG
Tfap2c	CACCGTGACCCCGATTGT	GAGTAATGGTCGGCGGACTG
Tpbpa	CCAGCACAGCTTTGGACATCA	AGCATCCAACTGCGCTTCA
